# WILKIE'S SYNDROME: A RARE CAUSE OF INTESTINAL OBSTRUCTION

**DOI:** 10.1590/0102-6720201600010020

**Published:** 2016

**Authors:** Ayşe KEFELI, Adem AKTÜRK, Bora AKTAŞ, Kerim ÇALAR

**Affiliations:** Kecioren Training Hospital, Gastroenterology Deparment, Pınarbası St. Sanatoryum Av. No:25 Kecioren and Siirt State Hospital, Radiology Department Abdullah Özgür Yeniova Gaziosmanpaşa University, Gastroenterology Department, Ankara,Turkey

## INTRODUCTION

Superior mesenteric artery (SMA) syndrome or Wilkie's syndrome is a rare but potentially life threatening gastrointestinal condition. This syndrome is a clinical phenomenon believed to be caused by compression of the third part of the duodenum between the SMA and the aorta, leading to obstruction. Patients may present symptoms of gastrointestinal obstruction, such as with recurrent episodes vomiting, upper abdominal distension and epigastric tenderness^8^. Various etiology theories, clinical course and treatment options have hitherto been discussed^5^. An interdisciplinary teamwork provides the most beneficial diagnostic and therapeutic result in this often underestimated disease.

## CASE REPORT

**FIGURE 1:**
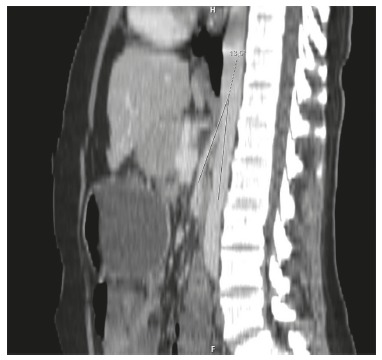
- CT of the abdomen showing reduced angle between the superior mesenteric artery and the aorta, with compression of the duodenum

A 27 years old woman was referred to our hospital, with recurrent episodes of profuse vomiting and upper abdominal pain associated with loss of appetite and dyspepsia since two years. She had no other comorbidities. Had been treated at another hospital with proton pump inhibitors, analgesics and intravenous fluids. She had a history of chronic anorexia and progressive loss of weight along with recurrent episodes of vomiting and upper abdominal pain. Clinical examination revealed dehydration, asthenicity (body mass index 19,5 kg/m^2^, weight: 50 kg, length:160 cm), abdominal distension, epigastric tenderness. Laboratory investigations showed a total white cell count of 9 500 mm^3^ and hypokalaemia (serum potassium: 3 mEq/l). Plain radiograph of the abdomen revealed gastric dilation. Ultrasonography was unremarkable. Upper gastrointestinal endoscopy showed dilated stomach and duodenum. Contrast-enhanced *computerized tomography* scan revealed grossly distended stomach and duodenum proximal to the third part of the duodenum at the level of the origin of superior mesenteric artery with abrupt narrowing at this level, suggestive of Wilkie's syndrome. While, normally, the angle between the SMA and the aorta is 22° to 60°, in this case, the aortomesenteric angle was 13,5°(Figure 1). In this case, conservative management was inefficient, so surgical treatment aiming to bypass the obstruction by an anastomosis between the jejunum and the proximal duodenum (duodenojejunostomy) was successful.

## DISCUSSION

Wilkie's syndrome occurs when the third portion of the duodenum is compressed between the SMA and the aorta. While, normally, the angle between the SMA and the aorta is 25° to 60°, it is narrowed in this syndrome^7^. The aortomesenteric angle may be narrowed because of congenital anomalies, significant weight loss, lumbar hyperlordosis, restorative proctocolectomy with ileal-anal anastomosis^1^^,^ 2 ^,^ 6. Clinical features of Wilkie's syndrome are entirely vague and non-specific. The most prominent symptoms are post-prandial abdominal pain (59%), nausea (40%), vomiting (50%), early satiety (32%), and anorexia (18%). These symptoms are aggravated by lying supine after eating and are relieved by assuming the left lateral decubitus, prone or knee-chest position^3^. These symptoms are compatible with more common conditions such as peptic ulcer disease, biliary colic, pancreatitis, and mesenteric ischemia. Physical examination generally reveals an asthenic body habitus. 

The diagnosis of Wilkie's Syndrome requires a high degree of clinical suspicion confirmed by radiographic studies demonstrating compression of the third portion of the duodenum. CT of the abdomen typically shows gastric and duodenal dilation and narrowed aortomesenteric angle^9^. Wilkie's syndrome responds to conservative management in the form of adequate nutrition by enteral/parenteral feeding and proper positioning of the patient after feeds. Surgery is resorted to when conservative measures are ineffective or in patients with long history of progressive weight loss or pronounced duodenal dilatation with stasis and complications^4^.

## References

[1] Adson DE, Mitchell JE, Trenkner SW (1997). The superior mesenteric artery syndrome and acute gastric dilatation
in eating disorders: a report of two cases and a review of the
literature. Int J Eat Disord.

[2] Goitein D, Gagne DJ, Papasavas PK, Dallal R, Quebbemann B, Eichinger JK, Johnston D, Caushaj PF (2004). Superior mesenteric artery syndrome after laparoscopic Roux-en-Y
gastric bypass for morbid obesity. Obes Surg.

[3] Hines JR, Gore RM, Ballantyne GH (1984). Superior mesenteric artery syndrome. Diagnostic criteria and
therapeutic approaches. Am J Surg.

[4] Massoud WZ (1995). Laparoscopic management of superior mesenteric artery
syn-drome. Int Surg.

[5] Mathenge N, Osiro S, Rodriguez II, Salib C, Tubbs RS, Loukas M (2014). Superior mesenteric arterysyndrome and its associated gastrointestinal
implications. Clin Anat.

[6] Matheus Cde O, Waisberg J, Zewer MH, Godoy AC (2005). Syndrome of duodenal compression by the superior mesenteric artery
following restorative proctocolectomy: a case report and review of
literature. Sao Paulo Med J.

[7] Neri S, Signorelli SS, Mondati E, Pulvirenti D, Campanile E, Di Pino L, Scuderi M, Giustolisi N, Di Prima P, Mauceri B, Abate G, Cilio D, Misseri M, Scuderi R (2005). Ultrasound imaging in diagnosis of superior mesenteric artery
syndrome. J Intern Med.

[8] Shiu JR, Chao HC, Luo CC, Lai MW, Kong MS, Chen SY, Chen CC, Wang CJ (2010). Clinical and nutritional outcomes in children with idiopathic superior
mesenteric artery syndrome. J Pediatr Gastroenterol Nutr.

[9] Unal B, Aktas A, Kemal G, Bilgili Y, Güliter S, Daphan C, Aydinuraz K (2005). Superior mesenteric artery syndrome: CT and ultrasonography
findings. Diagn Interv Radiol.

